# Carboxylic acid accumulation and secretion contribute to the alkali-stress tolerance of halophyte *Leymus chinensis*

**DOI:** 10.3389/fpls.2024.1366108

**Published:** 2024-03-19

**Authors:** Huan Wang, Shuting Zhao, Bo Sun, Feisal Mohamed Osman, Zexin Qi, Dan Ding, Xin Liu, Jiale Ding, Zhian Zhang

**Affiliations:** Department of Agronomy, Jilin Agricultural University, Changchun, China

**Keywords:** *Leymus chinensis*, metabolomic, root exudate, carboxylic group, stress

## Abstract

*Leymus chinensis* is a dominant halophytic grass in alkalized grasslands of Northeast China. To explore the alkali-tolerance mechanism of *L. chinensis*, we applied a widely targeted metabolomic approach to analyze metabolic responses of its root exudates, root tissues and leaves under alkali-stress conditions. *L. chinensis* extensively secreted organic acids, phenolic acids, free fatty acids and other substances having -COOH or phosphate groups when grown under alkali-stress conditions. The buffering capacity of these secreted substances promoted pH regulation in the rhizosphere during responses to alkali stress. *L. chinensis* leaves exhibited enhanced accumulations of free fatty acids, lipids, amino acids, organic acids, phenolic acids and alkaloids, which play important roles in maintaining cell membrane stability, regulating osmotic pressure and providing substrates for the alkali-stress responses of roots. The accumulations of numerous flavonoids, saccharides and alcohols were extensively enhanced in the roots of *L. chinensis*, but rarely enhanced in the leaves, under alkali-stress conditions. Enhanced accumulations of flavonoids, saccharides and alcohols increased the removal of reactive oxygen species and alleviated oxygen damage caused by alkali stress. In this study, we revealed the metabolic response mechanisms of *L. chinensis* under alkali-stress conditions, emphasizing important roles for the accumulation and secretion of organic acids, amino acids, fatty acids and other substances in alkali tolerance.

## 1 Introduction

Soil alkalization is a global environmental concern, and it has caused severe problems in some areas of the world, such as Northeast China. The primary causes of soil alkalization in this area are the presence of Na_2_CO_3_ and NaHCO_3_, resulting in a pH level above 10 in soils (Yin et al., 2017). More than 50% of the grasslands in this region are currently at risk of soil alkalization (Ran et al., 2020), and only a few alkali-tolerant halophytes can survive under such alkaline conditions. Understanding the mechanisms of alkali-tolerant halophytes will be useful for addressing the existing issue in alkalized grasslands. Alkali stress affects plants through osmotic regulation, ion toxicity and high pH, as well as resulting in indirect oxidative stress (Wang et al., 2012; Rao et al., 2013; Cao et al., 2022). High pH levels caused by alkali stress can significantly disrupt the pH stability of plant cells and compromise the integrity of cell membranes (Fang et al., 2021). Additionally, a high pH can lead to the precipitation of mineral nutrient ions in the rhizosphere soil (Wang et al., 2012). Therefore, for plants to survive in alkalized soil, it is crucial to adjust the pH value of the rhizosphere (Li et al., 2022).

Recently, increased attention has been given to alkali stress tolerance of plants (Cao et al., 2022), especially that of glycophytes, such as Arabidopsis (Yang et al., 2019), maize (Cao et al., 2020), rice, sorghum and millet (Zhang et al., 2023). In Arabidopsis and maize, H^+^-ATPases play important roles in alkali tolerance (Yang et al., 2019). In rice, sorghum, millet and maize (Zhang et al., 2023), an atypical G protein gene (*AT1*) affects the phosphorylation of aquaporins to modulate the distribution of H_2_O_2_, a form of reactive oxygen species caused by alkali stress. Halophytes and glycophytes may employ different mechanisms to resist salt or alkali stress (Rozema and Schat, 2013; Yuan et al., 2016). For example, leaves of halophytes can generally tolerate high Na^+^ concentrations or mitigate Na^+^-related damage via salt-secreting glands (Flowers and Colmer, 2008), whereas glycophytes, such as wheat, can limit influxes of Na^+^ into aboveground organs by extracting Na^+^ into roots (Wang and Xia, 2018). Thus, gaining a comprehensive understanding of the metabolic regulatory mechanisms responsible for alkali tolerance in halophytes will aid in improving crop alkali tolerance (Himabindu et al., 2016).

Root secretions play important roles in plant–microorganism interactions and in surviving abiotic stress conditions. The regulation of acidic substances (e.g., organic acids) is particularly crucial in this process. Root secretions containing organic acids play important roles in the detoxification of plants under heavy-metal stress (Fu et al., 2018; Awasthi et al., 2019). Moreover, plants also secrete acidic substances in response to abiotic stresses like drought (Ulrich et al., 2022), alkali stress (Yang et al., 2010; Guo et al., 2018) and low phosphorus stress (Tiziani et al., 2020). Under alkali-stress conditions, plants need to regulate the pH value outside the roots to maintain their function. The secretion of organic acids by roots is strongly associated with the alkali tolerance of plants, and it plays a crucial role in regulating cell and rhizosphere pH levels (Xiang et al., 2019; Li et al., 2022). For example, alkali stress increases secretion rates of oxalate, tartrate, malate, acetate and citrate in grape plants (Guo et al., 2018), and it also elevates secretion rates of oxalate, formate, lactate and acetate in *Chloris virgata* (Yang et al., 2010).

*Leymus chinensis* is the dominant forage grass in alkalized grasslands of Northeast China. It exhibits a remarkable ability to survive even under alkali-stress condition of 175 mM Na_2_CO_3_ (pH>11) (Chen et al., 2019). *L. chinensis* is an important forage grass because of its high productivity, high protein content and good palatability (Lin et al., 2016; Zhao et al., 2016; Chen et al., 2019). Its wide adaptability and economic value make it an ideal species for the restoration of alkaline lands in Northeast China. Although several studies have focused on physiological characteristics, metabolism and transcriptional changes of *L. chinensis* under alkali stress (Jin et al., 2008; Yan et al., 2022, 2023), most of them are descriptive. There is a lack of research on the metabolic regulation of different organs and the root secretions of *L. chinensis* grown under alkali-stress conditions. Here, a widely targeted metabolome technology, using ultra high-performance liquid chromatography/stem mass spectroscopy (UPLC-MS/MS) and real standard samples, were used to qualify and quantify metabolites in leaves, roots and root exudates of *L. chinensis* under alkali-stress conditions. The metabolic regulatory mechanisms and root secretion response processes of *L. chinensis* were investigated under alkali stress. The findings of this research will improve our understanding of plant alkali tolerance and contribute to the restoration and exploration of alkaline lands.

## 2 Materials and methods

### 2.1 Plant materials

The test organism used in this study was *L. chinensis* variety ‘Jingmu-3’, which was provided by the Heilongjiang Academy of Agricultural Sciences. It is not a nationally protected plant, and therefore, no special permission was required for its use in this research.

### 2.2 Plant cultivation

The experiment was conducted at Jilin Agricultural University (125.42° E, 43.81° N), Changchun, China. *L. chinensis* seeds (8 per pot) were sown in pots filled with clean sand and cultured using Hoagland nutrient solution for 30 days before being exposed to alkali-stress treatments. The control group was cultured with Hoagland nutrient solution, whereas the alkali-stress group (8 plants/pot) was irrigated thoroughly once a day with Hoagland nutrient solution containing 180 mM alkaline salt (molar ratio NaHCO_3_: Na_2_CO_3 = _9:1, pH 8.7). All the pots were placed in a greenhouse (23–25°C day and 17–20°C night, 16 h light). After 2 days of treatment, the mature leaves and roots of *L. chinensis* (tillering stage) were harvested and stored at −80°C for a metabolomics analysis. The root exudates were collected using Li et al.’s method in which root exudates were collected by rinsing sand with distilled water (Li et al., 2022). The collected exudates were then stored at −80°C for a metabolomics analysis. Each treatment had three biological replicates, with each biological replicate being a pool of all the plants or root exudates from a pot.

### 2.3 Metabolomics analysis

Relative concentrations of metabolites were quantified using a widely targeted metabolomics approach based on local MS/MS data library constructed with authentic standards (Chen et al., 2013, 2020). A mixed sample of equal volume of all extracts was loaded into the QTRAP system to construct a MS2 spectral tag library. Retention time, m/z ratio and fragmentation information were used to identify each metabolite by matching to the in-house database MWDB (https://www.metware.cn), and all the identified metabolites were quantified using a MRM method (Chen et al., 2013, 2020). The full scan investigation of MS/MS data was continuously evaluated using Analyst 1.6.3 software (AB Science, Waltham, MA, USA) to collect and trigger the acquisition of MS/MS spectra.

### 2.4 Statistical analyses

Statistical analyses were performed using SPSS (IBM Ltd, Armonk, V). Images were created using TBtools (Chen et al., 2020), GraphPad Prism 9 (Swift, 1997) and Visio 2021 (Microsoft Ltd, Redmond, USA). A KEGG enrichment analysis of differentially accumulated metabolites (DAMs) was conducted using the OmicShare tool (https://www.omicshare.com/tools). DAMs or differentially secreted metabolites (DSMs) under control and alkali-stress conditions were defined using the following criteria: VIP > 1, *P*-value (*t*-test) < 0.05 and |log2(Fold change)| > 1.

## 3 Results

### 3.1 Metabolic changes in root exudates

**Figure 1:**
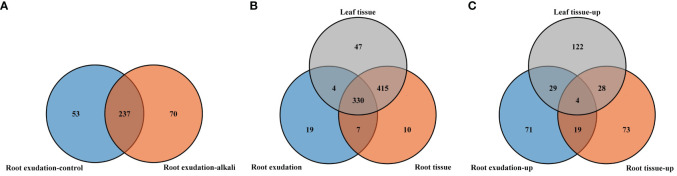
Venn diagram showing the number of detected metabolites in root exudates, root tissues and leaf tissues of *L. chinensis*. **(A)** Comparison of all the identified root exudates from plants grown under control and alkali-stress conditions. **(B)** Comparison of the numbers of the detected metabolites in root exudates, root tissues and leaf tissues. **(C)** Comparison of the numbers of upregulated metabolites in root exudates, root tissues and leaf tissues.

**Figure 2:**
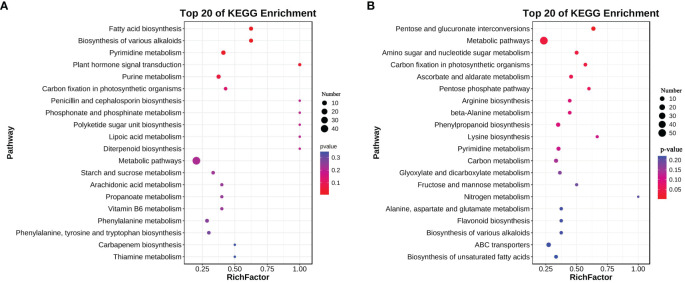
KEGG enrichment of upregulated metabolites in root exudates **(A)** and roots **(B)** and of *L. chinensis* after expose to alkali stress.

A total of 360 metabolites were detected in root exudates of *L. chinensis*, including 67 free fatty acids, 50 phenolic acids, 42 organic acids, 40 flavonoids, 34 saccharides or alcohols, 32 lipids, 31 amino acids or amino acid derivatives, 28 nucleotide or nucleotide derivatives, 20 alkaloids, 8 vitamins, 5 terpenoids, 2 lignans or coumarins and 1 tannin (**Supplementary Figure 1** and **Supplementary Table 1**). A total of 290 root exudates were detected under control conditions, whereas 307 root exudates were detected under alkali-stress conditions (**Figure 1A**). We particularly focused on root exudates having upregulated secretion rates under alkali-stress conditions (**Supplementary Table 2**). Seventy root exudates were detected in alkali-stressed plants but not in control plants, including 20 phenolic acids, 9 amino acids or amino acid derivatives, 8 organic acids, 7 nucleotides and derivatives, 7 flavonoids, 6 saccharides or alcohols, 4 free fatty acids, 4 alkaloids, 2 terpenoids, 1 lipid, 1 lignin or coumarin and 1 vitamin (**Figure 1A**, **Supplementary Tables 3**, **4**). We detected 123 upregulated root exudates (VIP > 1, *P*-value (*t*-test) < 0.05, |log2(Fold change)| > 1), including 30 phenolic acids, 23 free fatty acids, 16 organic acids, 15 nucleotides and nucleotide derivatives, 11 amino acids or amino acid derivatives, 10 saccharides or alcohols, 6 alkaloids, 5 flavonoids, 2 lignins and coumarins, 2 vitamins, 2 terpenoids and 1 lipid. Among these upregulated root exudates, 63 contained -COOH groups, including 23 free fatty acids, 14 organic acids, 12 phenolic acids, 11 amino acids and derivatives, 1 alkaloid, 1 lignan or coumarin and 1 vitamin (**Supplementary Tables 2** and **5**). We performed a KEGG enrichment analysis on the upregulated root exudates and found that they were significantly enriched in fatty acid biosynthesis, biosynthesis of various alkaloids, and pyrimidine metabolism (*P* < 0.05) (**Figure 2A**).

### 3.2 Metabolic changes in roots

We collectively detected 762 metabolites in the roots, which included 131 phenolic acids, 119 flavonoids, 87 lipids, 75 free fatty acids, 74 organic acids, 68 alkaloids, 55 amino acids or amino acid derivatives, 42 saccharides and alcohols, 39 nucleotides and derivatives, 26 lignans or coumarins, 18 terpenoids, 15 vitamins, 10 others and 3 tannins (**Supplementary Figure 1B**). We focused on metabolites with upregulated levels induced by alkali stress (VIP > 1, *P*-value (*t*-test) < 0.05, |log2(Fold change)| > 1). We detected 124 upregulated metabolites in the root tissue, including 22 flavonoids, 20 phenolic acids, 15 saccharides or alcohols, 13 organic acids, 11 free fatty acids, 11 alkaloids, 10 amino acids or amino acid derivatives, 10 nucleotides and derivatives, 5 lipids, 4 lignans or coumarins, 2 others and 1 vitamin (**Supplementary Table 2**). Of the 124 upregulated metabolites in root tissue, 40 metabolites contained -COOH groups, which included 11 organic acids, 10 free fatty acids, 10 amino acids or amino acid derivatives, 5 phenolic acids, 3 saccharides or alcohols and 1 nucleotide or derivative.

We performed a KEGG enrichment analysis on the 124 upregulated metabolites in roots and found that 66 metabolic pathways were enriched. These pathways included 13 carbohydrate metabolism pathways (e.g., pentose and glucuronate interconversions), 12 amino acid metabolism pathways (e.g., arginine biosynthesis), 10 biosynthesis pathways of other secondary metabolites, 6 lipid metabolism pathways (e.g., biosynthesis of unsaturated fatty acids), and 4 energy metabolism pathways (carbon fixation in photosynthetic organisms, nitrogen metabolism, oxidative phosphorylation and sulfur metabolism). Pentose and glucuronate interconversions, amino sugar and nucleotide sugar metabolism and carbon fixation in photosynthetic organisms were significantly enriched (*P* < 0.05) (**Figure 2B**).

### 3.3 Metabolic changes in leaves

A total of 796 metabolites were detected in leaves of *L. chinensis* under control and alkali-stress conditions. These metabolites included 149 phenolic acids, 141 flavonoids, 83 lipids, 74 organic acids, 70 free fatty acids, 68 alkaloids, 56 amino acids or amino acid derivatives, 41 saccharides or alcohols, 40 nucleotides and derivatives, 26 lignans or coumarins, 18 terpenoids, 15 vitamins, 5 tannins and 10 others (**Supplementary Figure 1C**). We focused on metabolites with upregulated levels under alkali-stress conditions (VIP > 1, *P*-value (*t*-test) < 0.05, |log2(Fold change)| > 1). In the leaves, we detected 183 upregulated metabolites, including 38 alkaloids, 29 lipids, 29 phenolic acids, 24 organic acids, 24 amino acids or amino acid derivatives, 14 nucleotides and derivatives, 10 free fatty acids, 5 saccharides or alcohols, 4 vitamins, 3 flavonoids, 1 terpenoid and 2 others (**Supplementary Table 2**). Among these 183 upregulated leaf metabolites, 76 metabolites contained -COOH groups, including 24 amino acids or amino acid derivatives, 23 organic acids, 15 phenolic acids, 9 free fatty acids, 2 alkaloids, 2 vitamins and 1 nucleotide or derivative.

**Figure 3:**
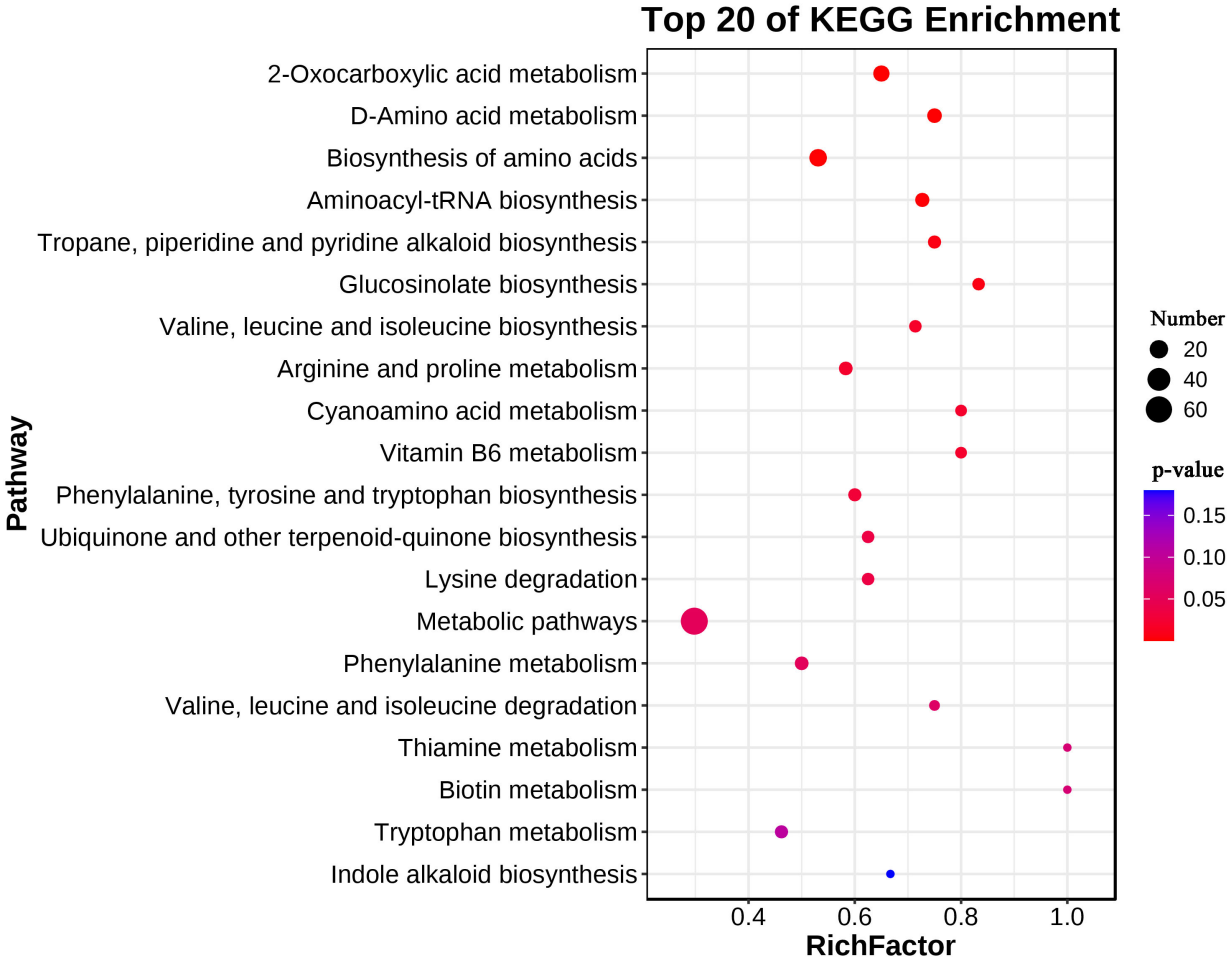
KEGG enrichment of upregulated metabolites in leaves of *L. chinensis* after expose to alkali stress.

A KEGG enrichment analysis was performed on 183 upregulated metabolites in the leaves. A total of 75 metabolic pathways were enriched, which included 14 biosynthesis of other secondary metabolites, 13 amino acid metabolism pathways, 12 carbohydrate metabolism pathways (e.g., fructose and mannose metabolism), 7 lipid metabolism pathways (e.g., fatty acid degradation) and 3 energy metabolism pathways (nitrogen metabolism, sulfur metabolism and carbon fixation in photosynthetic organisms). The upregulated metabolites in the leaves were significantly enriched in 2-oxocarboxylic acid metabolism, D-amino acid metabolism, biosynthesis of amino acids, aminoacyl-tRNA biosynthesis, tropane, piperidine and pyridine, alkaloid bio-synthesis, glucosinolate biosynthesis, valine, leucine and isoleucine biosynthesis, arginine and proline metabolism, cyanoamino acid metabolism, vitamin B6 metabolism, phenylalanine, tyrosine and tryptophan biosynthesis, ubiquinone and other terpenoidquinone biosynthesis and lysine degradation (*P* < 0.05) (**Figure 3**).

### 3.4 Comparison of metabolic responses in root exudates, roots and leaves

**Figure 4:**
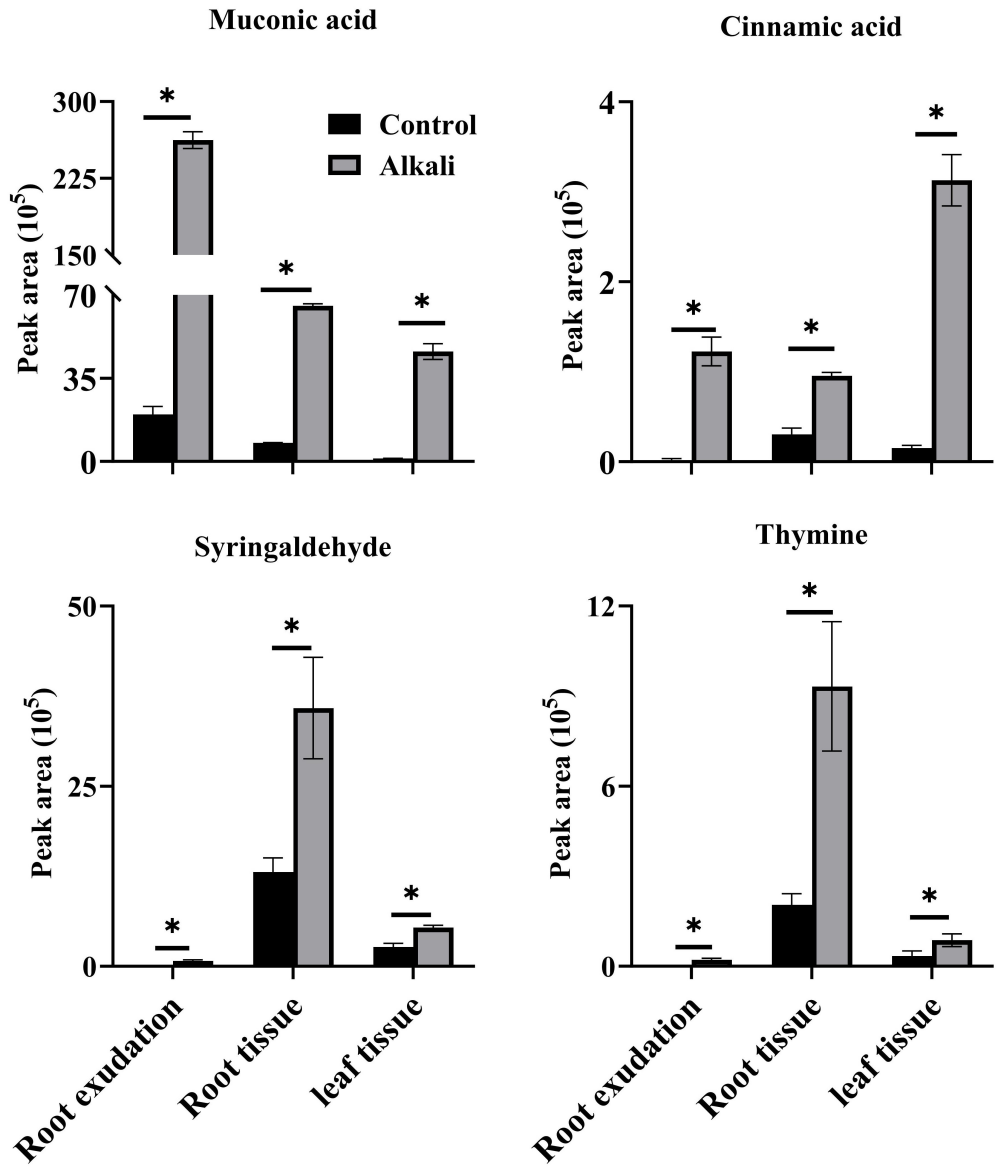
Shared upregulated metabolites in root exudates, root tissues and leaf tissues of *L. chinensis.* The asterisks (*) indicate significant differences among treatments.

**Figure 5:**
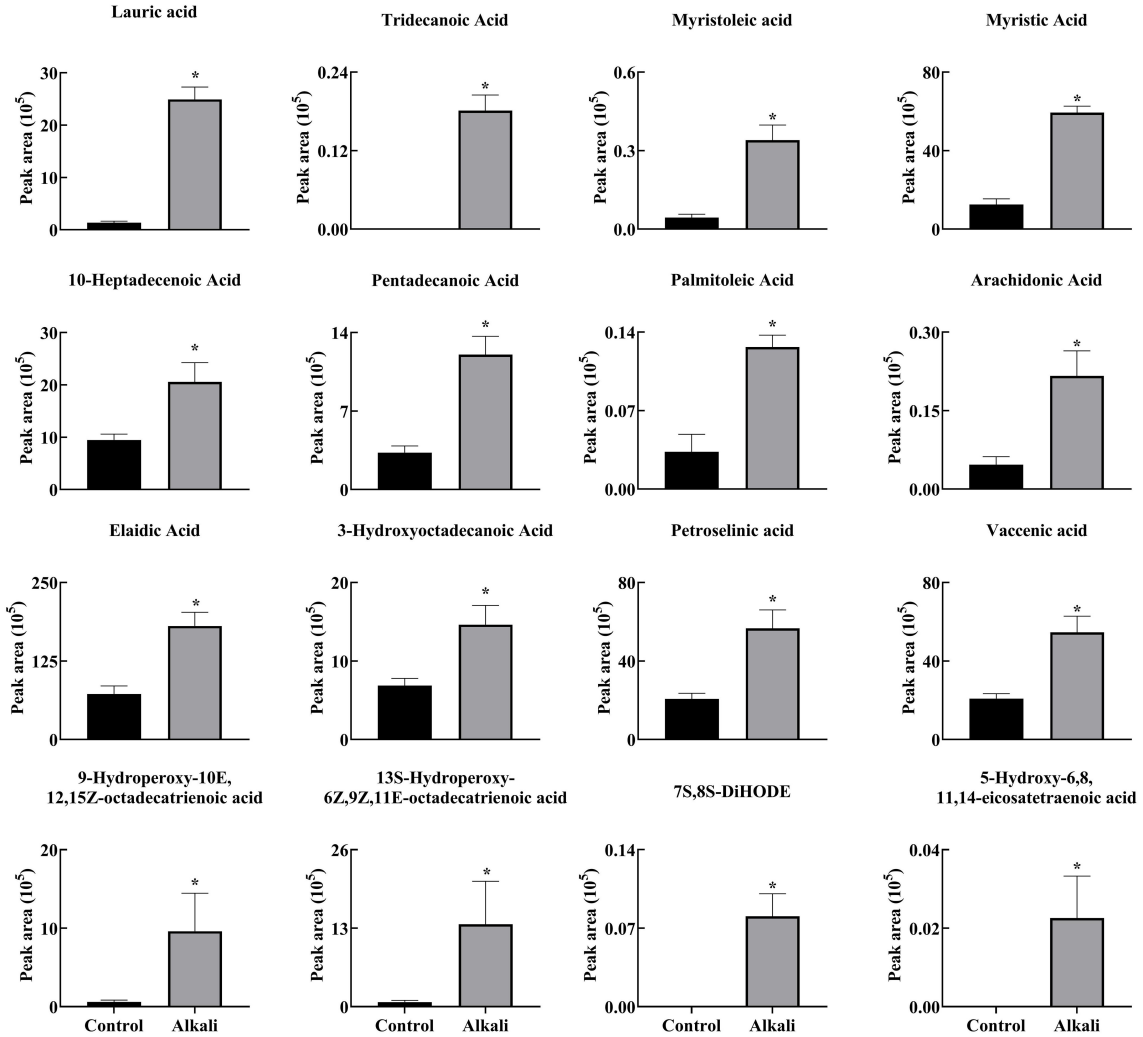
Effects of alkali stress on the secretion rates of free fatty acids in *L. chinensis*. The levels of the metabolites were upregulated only in root exudates, not in roots or leaves. The asterisks (*) indicate significant differences between treatments.

**Figure 6:**
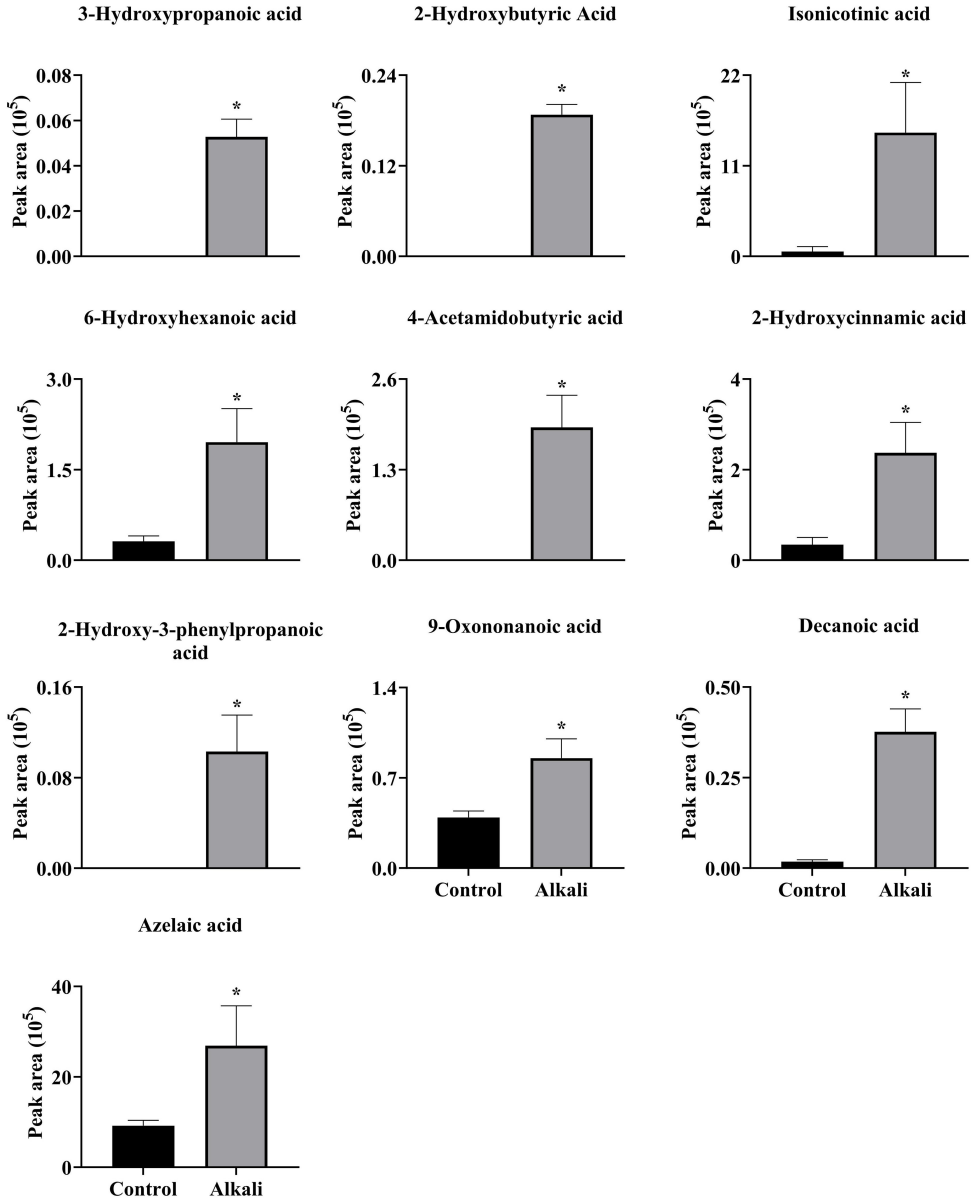
Effects of alkali stress on secretion rates of organic acids in *L. chinensis*. The levels of the metabolites were upregulated only in root exudates, not in roots or leaves. The asterisks (*) indicate significant differences between treatments.

**Figure 7:**
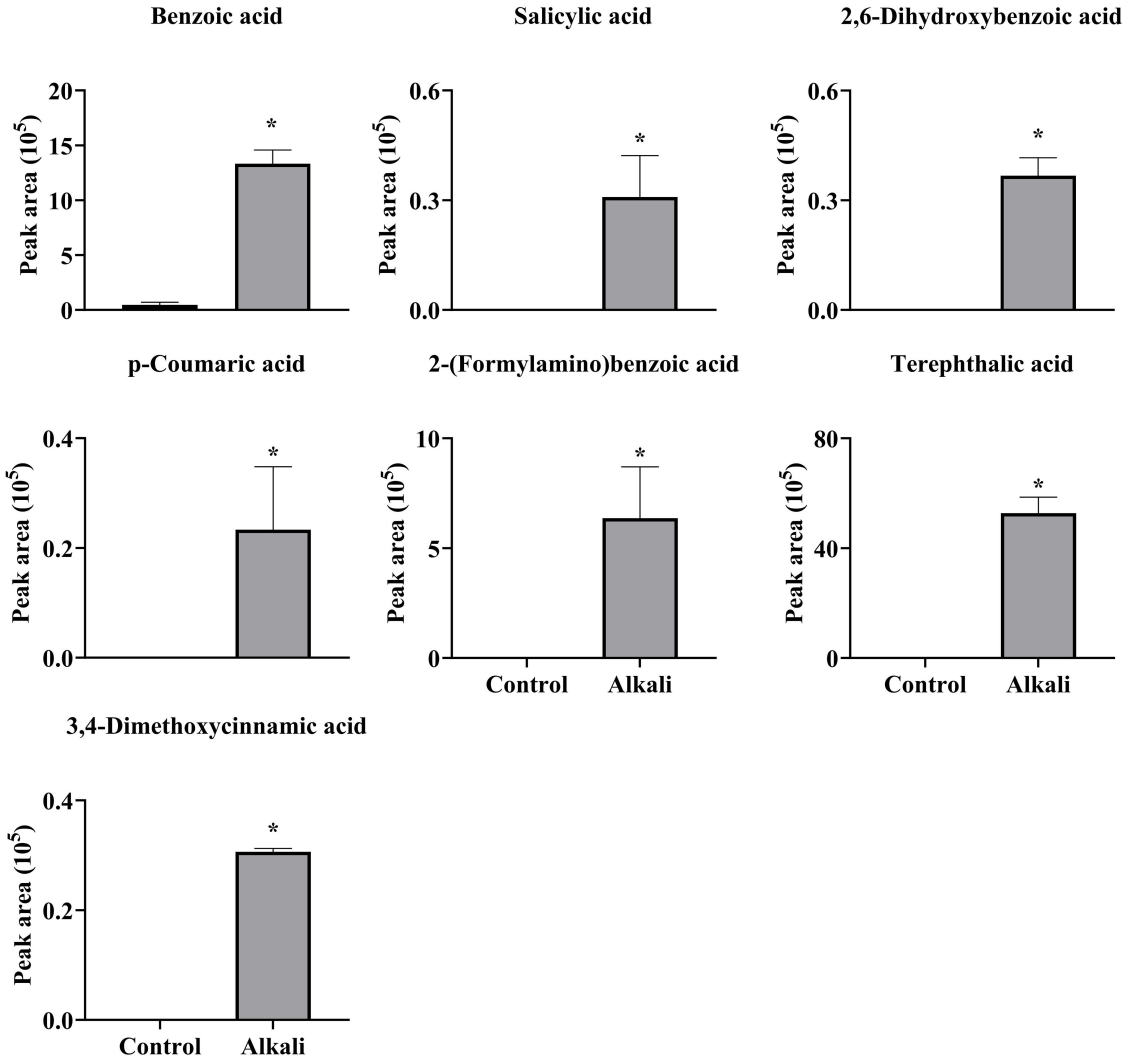
Effects of alkali stress on secretion rates of phenolic acids containing -COOH in *L. chinensis*. The levels of the metabolites were upregulated only in root exudates, not in roots or leaves. The asterisks (*) indicate significant differences between treatments.

**Figure 8:**
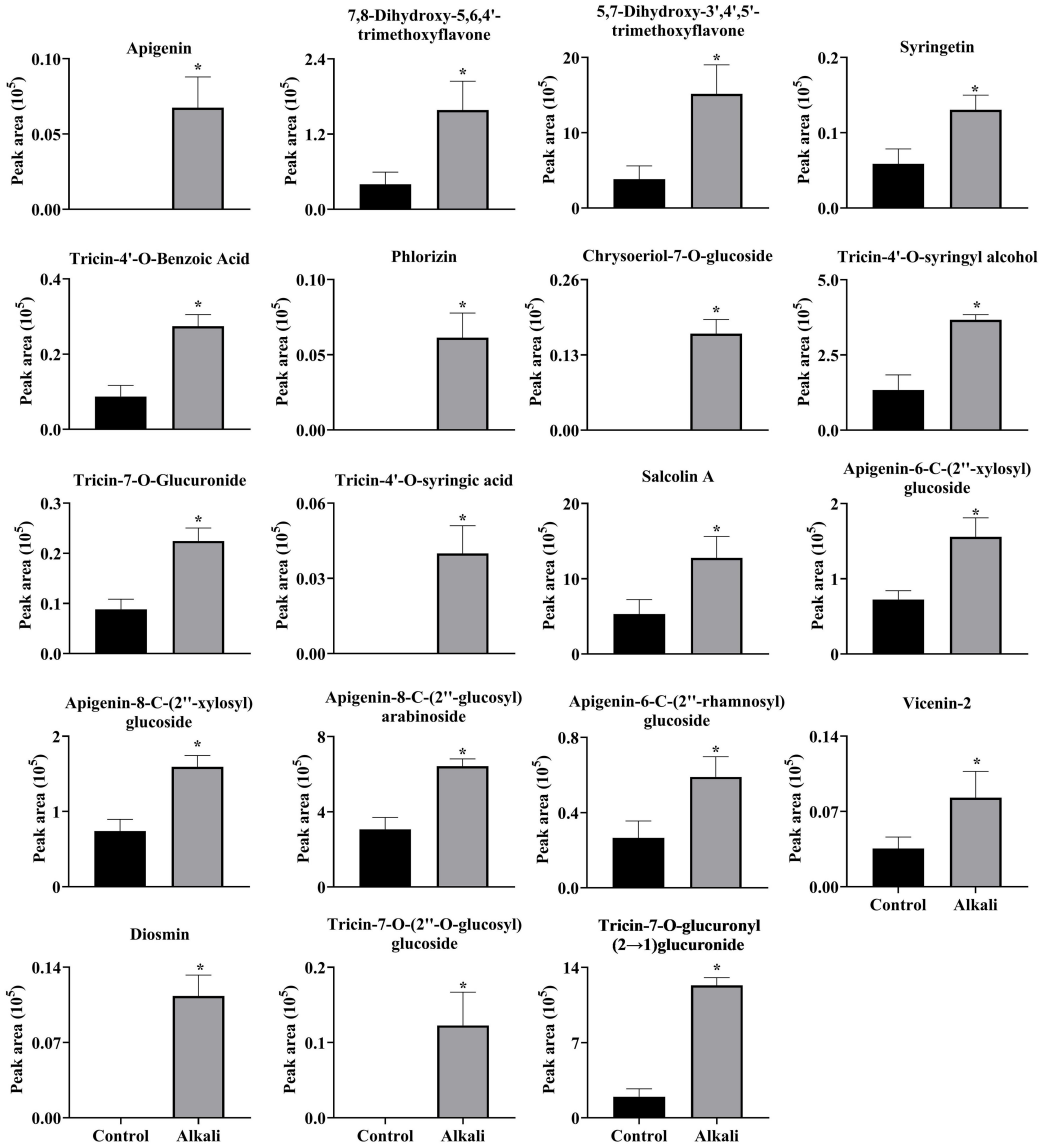
Effects of alkali stress on accumulations of flavonoids in roots of *L. chinensis*. The levels of the metabolites were upregulated only in roots, not in root exudates or leaves. The asterisks (*) indicate significant differences between treatments.

**Figure 9:**
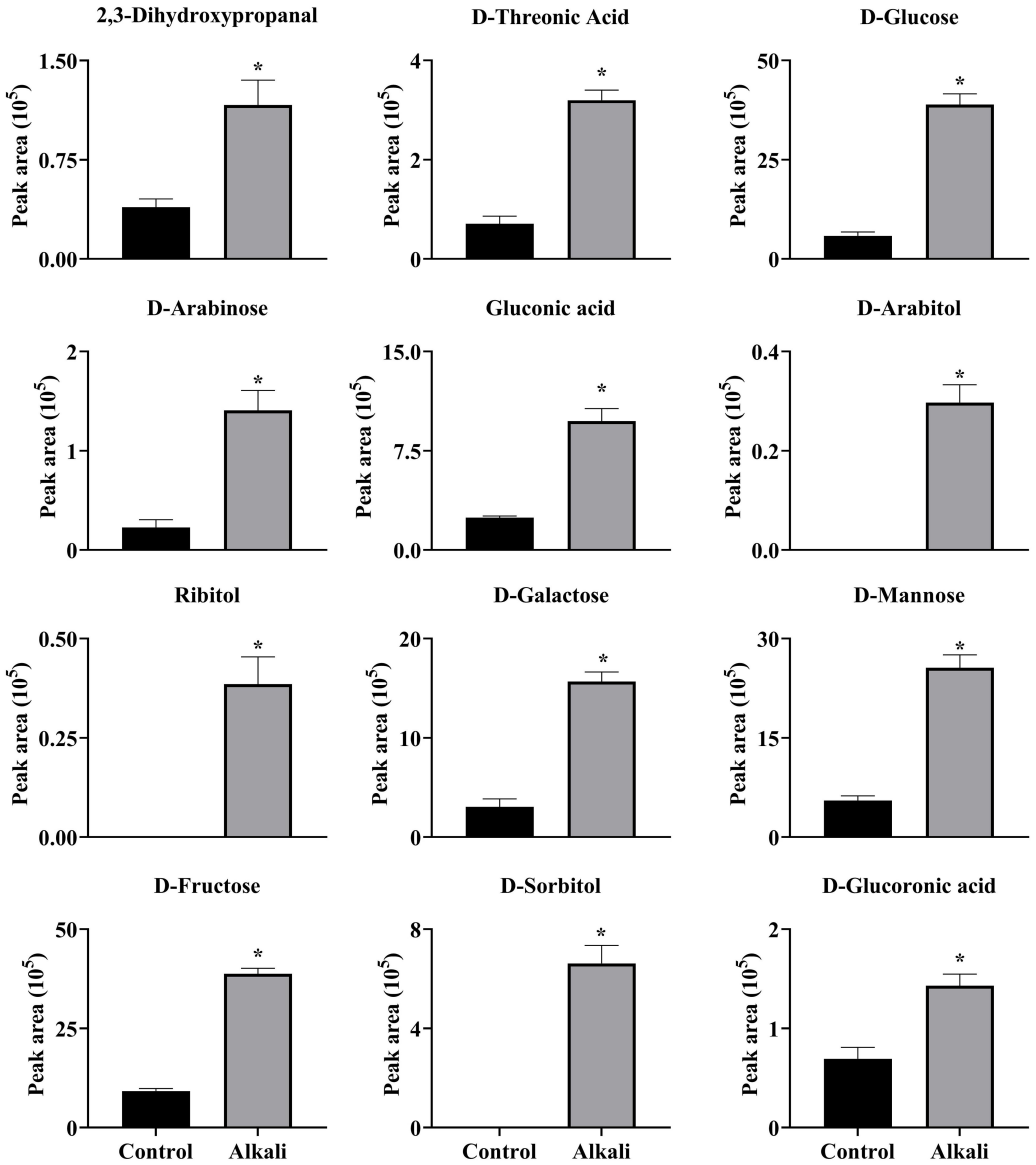
Effects of alkali stress on accumulations of saccharides and alcohols in roots of *L. chinensis*. The levels of the metabolites were upregulated only in roots, not in root exudates or leaves. The asterisks (*) indicate significant differences between treatments.

**Figure 10:**
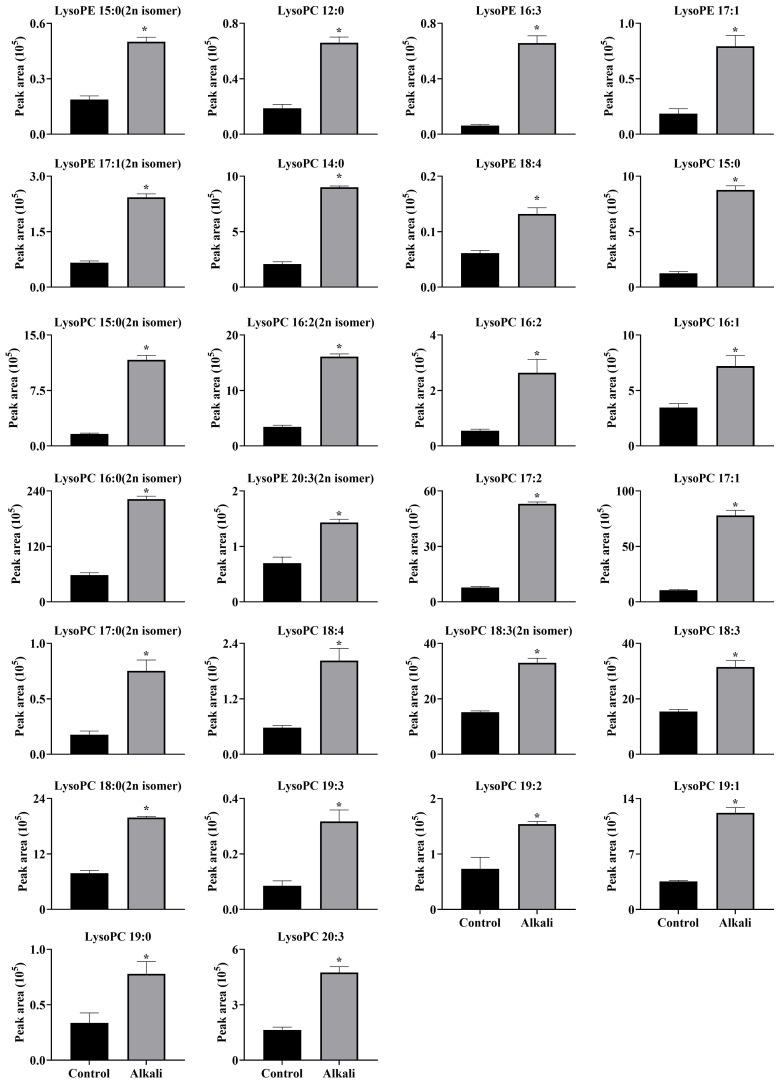
Effects of alkali stress on accumulations of glycerol phospholipids (lipids) in leaves of *L. chinensis*. The levels of the metabolites were upregulated only in leaves, not in root exudates or roots. The asterisks (*) indicate significant differences between treatments.

**Figure 11:**
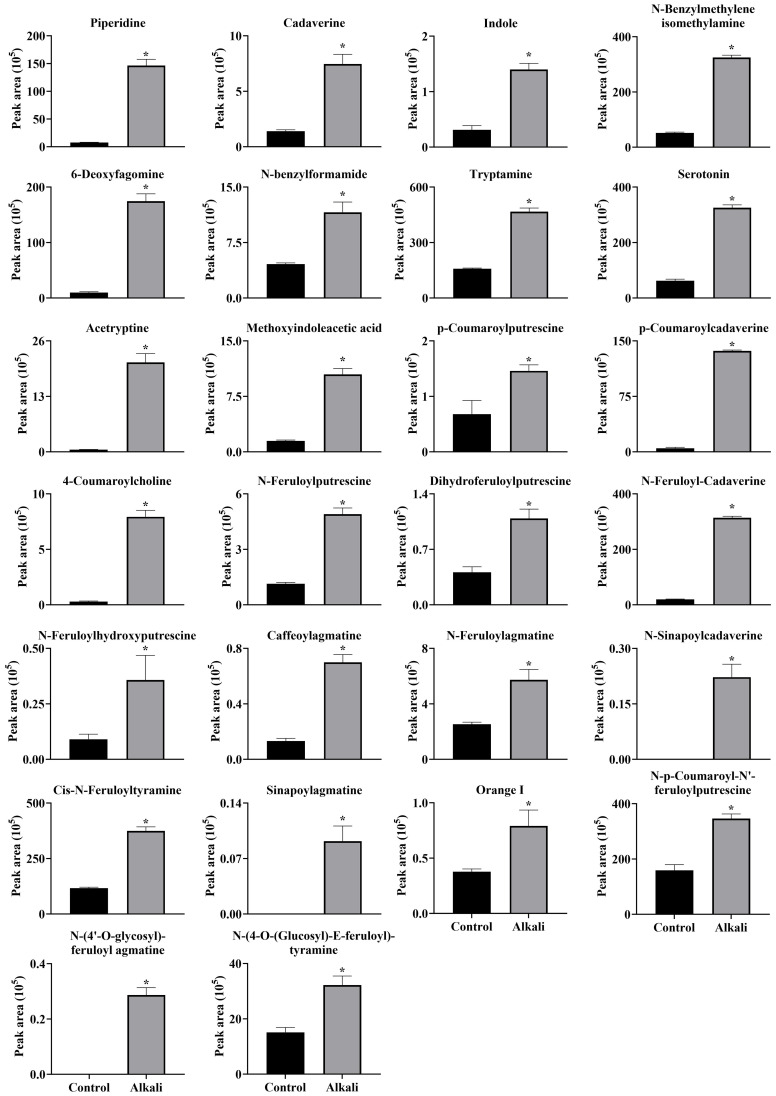
Effects of alkali stress on accumulations of alkaloids in leaves of *L. chinensis*. The levels of the metabolites were upregulated only in leaves, not in root exudates or roots. The asterisks (*) indicate significant differences between treatments.

**Figure 12:**
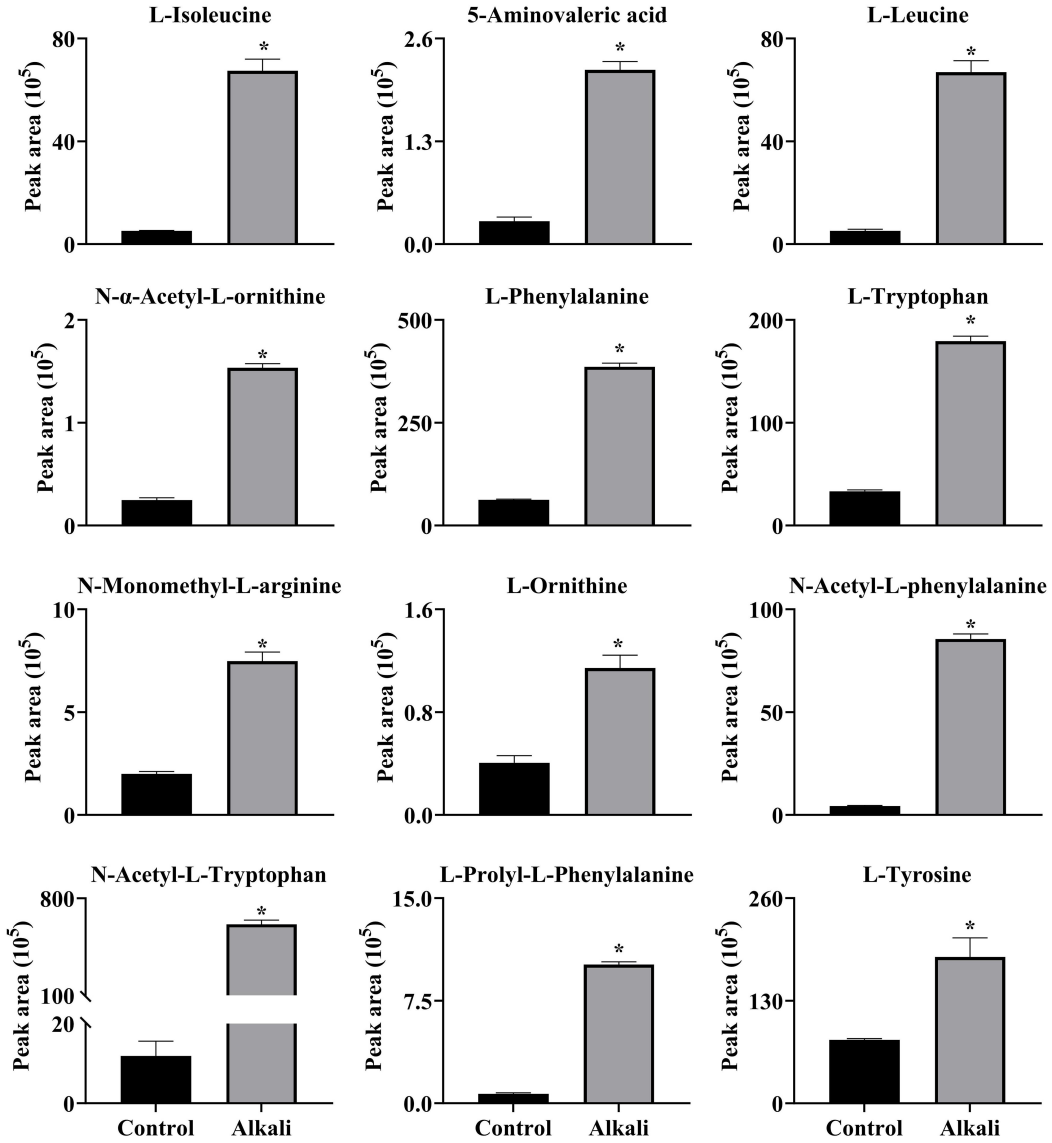
Effects of alkali stress on accumulations of amino acids and amino acid derivatives in leaves of *L. chinensis*. The levels of the metabolites were upregulated only in leaves, not in root exudates or roots. The asterisks (*) indicate significant differences between treatments.

We compared metabolite components of root exudates, root tissues and leaves of *L. chinensis*, and we identified 19, 10 and 47 metabolites unique to root exudates, root tissues and leaves, respectively (**Figure 1B**). We also compared components of metabolites with upregulated accumulations caused by alkali stress in root exudates, root tissues and leaf tissues (**Figure 1C**, **Supplementary Tables 5**-**7**), and we observed that four upregulated metabolites, muconic acid, cinnamic acid, syringaldehyde and thymine, were shared by root exudates, root tissues and leaf tissues (**Figure 4**). Among all the upregulated metabolites, 71 upregulated metabolites were unique to root exudates (not upregulated in roots or leaves), including16 free fatty acids (**Figure 5**), 10 organic acids (**Figure 6**), 18 phenolic acids (**Figure 7**), 7 nucleotides and derivatives, 7 saccharides or alcohols, 3 amino acids and derivatives, 3 flavonoids, 2 alkaloids, 2 lignans or coumarins, 1 lipid, 1 terpenoid and 1 vitamin (**Supplementary Table 5**). During the response of *L. chinensis* to alkali stress, 73 upregulated metabolites were unique to roots (not regulated in leaves or root exudates), including 19 flavonoids (**Figure 8**), 12 saccharides or alcohols (**Figure 9**), 8 phenolic acids, 7 organic acids, 6 amino acids or amino acid derivatives, 5 fatty acids, 5 nucleotides and derivatives, 4 lipids, 4 lignans or coumarins, 1 alkaloid, 1 vitamin and 1 other (**Supplementary Table 6**). After expose to alkali stress, 122 upregulated metabolites were unique to leaves (not regulated in roots or root exudates), including 26 glycerophospholipids (**Figure 10**), 26 alkaloids (**Figure 11**), 21 phenolic acids, 15 organic acids, 12 amino acid and derivatives (**Figure 12**), 6 nucleotides or derivatives, 5 free fatty acids, 3 saccharides or alcohols, 3 vitamins, 2 flavonoids, 2 glycerol esters and 1 other (**Supplementary Table 7**).

Additionally, root exudates and root tissues shared 19 upregulated metabolites, including 7 phenolic acids, 4 free fatty acids, 2 nucleotides and derivatives, 2 flavonoids, 2 saccharides or alcohols, 1 alkaloid and 1 organic acid. Five of these metabolites contained -COOH groups (**Supplementary Figure 2**). Leaves and roots shared 28 upregulated metabolites, including 9 alkaloids, 4 organic acids, 4 amino acids and derivatives, 3 phenolic acids, 2 free fatty acids, 2 nucleotides and derivatives, 1 flavonoid, 1 saccharide or alcohol, 1 lipid and 1 other compound. In total, 11 metabolites contained -COOH groups (**Supplementary Figure 3**). Furthermore, root exudates and leaves shared 29 upregulated metabolites, including 8 amino acids and derivatives, 5 nucleotides and derivatives, 4 organic acids, 3 alkaloids, 3 phenolic acids, 3 free fatty acids, 1 terpenoid, 1 sugar or alcohol and 1 vitamin. Among them, 18 metabolites contained -COOH groups (**Supplementary Figure 4**).

### 3.5 Metabolic pathway analyses of roots and leaves

Leaves and roots enhance the biosynthesis of amino acids, other secondary metabolites and carbohydrates to support growth, and root secretions are increased to adapt to alkali stress. In the energy metabolism pathway, *L. chinensis* roots and leaves had increased levels of fumaric, L-malic and D-erythrose-4-phosphate, with the enhancement being greater in roots than in leaves. After expose to alkali stress, the levels of 2-aminoethanesulfonic acid and D-glucose were increased only in the roots, whereas L-cysteine, citric acid and glycerone phosphate showed increased levels only in the leaves (**Supplementary Figure 5**). In the amino acid metabolism pathway, levels of L-cysteine, L-ornithine, L-leucine, L-tyrosine, L-tryptophan, L-phenylalanine and L-isoleucine were increased in the leaves but were decreased in the roots. Additionally, the level of L-aspartate was lower in the leaves but higher in the roots, indicating greater accumulations of amino acids in the leaves (**Supplementary Figure 6**). In the carbohydrate metabolism pathway, levels of D-glucose, D-mannose, D-glucoronic acid, D-sorbitol, D-galactose, D-arabitol, ribitol, D-arabinose and D-xylonic acid were increased in the roots but decreased in the leaves. Additionally, sorbitol-6-phosphate was found to be decreased in the roots but increased in the leaves, indicating that the roots tended to accumulate more carbohydrates (**Supplementary Figure 7**). For the biosynthesis of secondary metabolites pathway, roots exhibited higher levels of phlorizin, apigenin, chrysin and syringetin compared with leaves. Levels of piperidine and phenylpyruvate, but not cadaverine, were lower in roots, whereas other secondary metabolites showed enhanced accumulations in roots (**Supplementary Figure 8**).

## 4 Discussion

### 4.1 Alkali stress induced the secretion of phenolic acids, organic acids and fatty acids in *L. chinensis*

High soil pH can lead to the precipitation of various mineral element ions surrounding roots, reducing their bioavailability (Turner et al., 2020). High pH caused by alkali stress also affects the root morphology and functions of plants, consequently impacting above-ground symptoms (Turner et al., 2020). Therefore, plants that survive under alkali-stress conditions must adjust the pH of their rhizospheres. The application of organic acids immediately lowers the soil pH (Rukshana et al., 2014), and plants, such as grape hybrid rootstocks, seabuckthorn, *Chloris virgata*, wheat and grapevine, can mitigate the damage caused by alkali stress through the secretion of organic acids (Chen et al., 2009; Yang et al., 2010; Guo et al., 2018; Xiang et al., 2019; Li et al., 2020). Our previous study revealed that alkali stress strongly inhibits photosynthesis and growth of *L. chinensis*, and it disturbed the ion balance (Wang et al., 2020). Here, our results indicated that *L. chinensis* extensively secreted organic acids, phenolic acids, free fatty acids and other substances containing a -COOH or phosphate group after expose to alkali stress. The extensive secretion of these substances having a buffering capacity should promote pH regulation in the rhizosphere during the response of *L. chinensis* to alkali stress, allowing *L. chinensis* to exhibit a strong resistance to alkali stress. Additionally, during responses to alkali stress, *L. chinensis* had enhanced secretion rates for many metabolites without a -COOH or H_2_PO_4_ group, and this may play a crucial role in the interactions between *L. chinensis* roots and rhizosphere microorganisms that facilitate nutrient uptake. Alkali stress enhances the glycolysis level of *L. chinensis* roots by upregulating the expression of rate-limiting enzyme-encoding (6-phosphofructokinase and pyruvate kinase) genes (Wang et al., 2020). Glycolysis provides ATP, NADH, phosphoenolpyruvate and acetyl-CoA for metabolic processes and stress responses. Thus, enhanced glycolysis should support more energy and carbon sources for the alkali-stress responses of *L. chinensis* roots, including the accumulation and secretion of organic solutes. Li et al. also reported that alkali stress enhances the glycolysis level of halophyte *Puccinellia tenuiflora* roots by upregulating the expression of a rate-limiting enzyme-encoding (6-phosphofructokinase) gene to relieve the damage caused by alkali stress (Li et al., 2022). This suggests that enhanced glycolysis by upregulating rate-limiting enzyme-encoding genes may be a common response of halophytes to alkali stress. Genetic modifications of the rate-limiting glycolysis-related enzyme-encoding genes may be a promising strategy for improving the alkali tolerance of crop and forage grasses, and this should be investigated in depth.

### 4.2 Metabolic responses in roots and leaves

Alkali stress can strongly influence the metabolism of plants. For instance, grapevines subjected to alkali stress accumulate phenolic acids, flavonoids and alkaloids (Lu et al., 2023). Similarly, *Cinnamomum bodinieri* roots respond to alkali stress by accumulating alkaloids, flavonoids, N-acetyl-L-phenylalanine, lauric acid, palmitic acid, oxaloacetic acid and other substances to alleviate the negative effects of alkali stress (Han et al., 2023). Alkali stress triggers a noteworthy accumulation of nearly all free amino acids and ribose in mature wheat leaves, thereby enhancing tissue tolerance (Xiao et al., 2020). Similarly, the concentrations of amino acids, alkaloids, and organic acids in *Lycium barbarum* fruits are enhanced under salt-alkali stress (Liang et al., 2022). Hence, organic acids, amino acids and secondary metabolites significantly contribute to plant alkali-stress tolerance. Alkali stress specifically enhanced the accumulations of numerous flavonoids, saccharides and alcohols in the roots, but few in the leaves, of *L. chinensis*. Enhanced accumulations of flavonoids, saccharides and alcohols enables the more efficient removal of reactive oxygen species and the alleviation of oxygen damage caused by alkali stress. In addition, under alkali stress, *L. chinensis* leaves exhibited enhanced accumulations of free fatty acids, lipids, amino acids, organic acids, phenolic acids, and alkaloids, which should play important roles in maintaining cell membrane stability and osmotic regulation, as well as supporting the production of substrates for alkali-stress responses of the roots through long-distance transport. Additionally, there are 18 shared upregulated substances with a -COOH in the leaves and root exudates, but only 5 shared upregulated substances with a -COOH in the roots and root exudates. This indicated that leaves may be the main organs that provide carboxylic acid substances that are secreted by *L. chinensis* roots under alkali-stress conditions. The KEGG enrichment analysis revealed that the upregulated metabolites in both roots and leaves were associated with amino acid, secondary, carbohydrate and energy metabolism pathways. The enhancement of amino acid, carbohydrate and energy metabolism pathways in the leaves and roots facilitates the alkali-stress responses of *L. chinensis*. Alkali stress upregulates the expression of many key genes, including phenylalanine ammonia-lyase (rate-limiting enzyme for phenol synthesis) gene, 4-coumarate-CoA ligase gene, flavonoid 3’-monooxygenase gene, flavonoid 3’-monooxygenase gene, cinnamoyl-CoA reductase gene, and flavanone 3-dioxygenase gene involved in phenol (including phenolic acids and flavonoids) biosynthesis in leaves and roots of *L. chinensis* (Wang et al., 2020). These upregulated genes caused by alkali stress may enhance the synthesis of phenolic acids and flavonoids during responses of *L. chinensis* to alkali stress. Our results suggest that responses of plants to alkali stress require a complex network that coordinates numerous metabolic pathways, involving numerous genes. The genes (e.g., phenylalanine ammonia-lyase gene and 6-phosphofructokinase gene) involved in the synthesis of key metabolites and energy metabolism may form key nodes of the alkali-stress response network, but upstream regulatory genes may be effective targets for the genetic improvement of plant alkali tolerance. Molecular regulatory mechanisms of alkali tolerance should be explored in crops or model plants, with an emphasis on the upstream regulatory genes for key metabolites and energy metabolism. Our study provides useful information for further research on the molecular regulatory mechanisms of plant alkali tolerance.

## 5 Conclusions

The extensive secretion of substances having a buffering capacity promoted pH regulation of the rhizosphere during responses of *L. chinensis* to alkali stress. In the roots of *L. chinensis*, the enhanced accumulations of flavonoids, saccharides and alcohols enabled the more effective removal of reactive oxygen species and the alleviation of oxygen damage caused by alkali stress. *L. chinensis* leaves exhibited enhanced accumulations of free fatty acids, lipids, amino acids, organic acids, phenolic acids and alkaloids, which should play important roles in maintaining cell membrane stability and osmotic regulation, as well as supporting the production of substrates for alkali-stress responses in the roots.

## Data availability statement

The raw data supporting the conclusions of this article will be made available by the authors, without undue reservation.

## Author contributions

HW: Conceptualization, Funding acquisition, Resources, Investigation, Writing – original draft. SZ: Investigation, Writing7nbsp;– original draft, Data curation, Formal Analysis, Visualization. BS: Investigation, Visualization, Writing – original draft. FO: Investigation, Writing – original draft. ZQ: Investigation, Writing – original draft. DD: Data curation, Writing – original draft. XL: Data curation, Writing – original draft. JD: Formal Analysis, Writing – original draft. ZZ: Conceptualization, Funding acquisition, Methodology, Project administration, Resources, Supervision, Writing – review & editing.

## Supplementary material

Supplementary Figure 1 Classification of the metabolites detected in root exudates (A), root tissues (B) and leaf tissues (C) of *L. chinensis*.

Supplementary Figure 2 Comparison of the effects of alkali stress on the accumulations of metabolites in root exudates and root tissues of *L. chinensis*. The levels of the metabolites were upregulated in both root exudates and root tissues. The asterisks (*) indicate significant differences between treatments.

Supplementary Figure 3 Comparison of the effects of alkali stress on the accumulations of metabolites containing -COOH in roots and leaves of *L. chinensis*. The levels of the metabolites were upregulated in both roots and leaves. The asterisks (*) indicate significant differences between treatments.

Supplementary Figure 4 Comparison of the effects of alkali stress on the accumulations of metabolites containing -COOH in root exudates and leaves of *L. chinensis*. The levels of the metabolites were upregulated in both root exudates and leaves. The asterisks (*) indicate significant differences between treatments.

Supplementary Figure 5 Energy metabolism responses of root exudates, roots and leaves of *L. chinensis* under alkali-stress conditions. Red and green represent increased and decreased, respectively, accumulations of a metabolite. Yellow indicates that a metabolite was not detected. The asterisks (*) indicate significant differences between treatments.

Supplementary Figure 6 Amino acid metabolism responses of root exudates, roots and leaves of *L. chinensis* under alkali-stress conditions. Red and green represent increased and decreased, respectively, accumulations of a metabolite. Yellow indicates that a metabolite was not detected. The asterisks (*) indicate significant differences between treatments.

Supplementary Figure 7 Carbohydrate metabolism responses of root exudates, roots and leaves of *L. chinensis* under alkali-stress conditions. Red and green represent increased and decreased, respectively, accumulations of a metabolite. Yellow indicates that a metabolite was not detected. The asterisks (*) indicate significant differences between treatments.

Supplementary Figure 8 Secondary metabolite responses of root exudates, roots and leaves of *L. chinensis* under alkali-stress conditions. Red and green represent increased and decreased, respectively, accumulations of a metabolite. Yellow indicates that a metabolite was not detected. The asterisks (*) indicate significant differences between treatments.
