# Supplementary figures and images for: Carboxylic acid accumulation and secretion contribute to the alkali-stress tolerance of halophyte Leymus chinensis
Source: Front Plant Sci. 2024 Mar 19;15:1366108. doi: 10.3389/fpls.2024.1366108 (PMC10985159; doi:10.3389/fpls.2024.1366108)

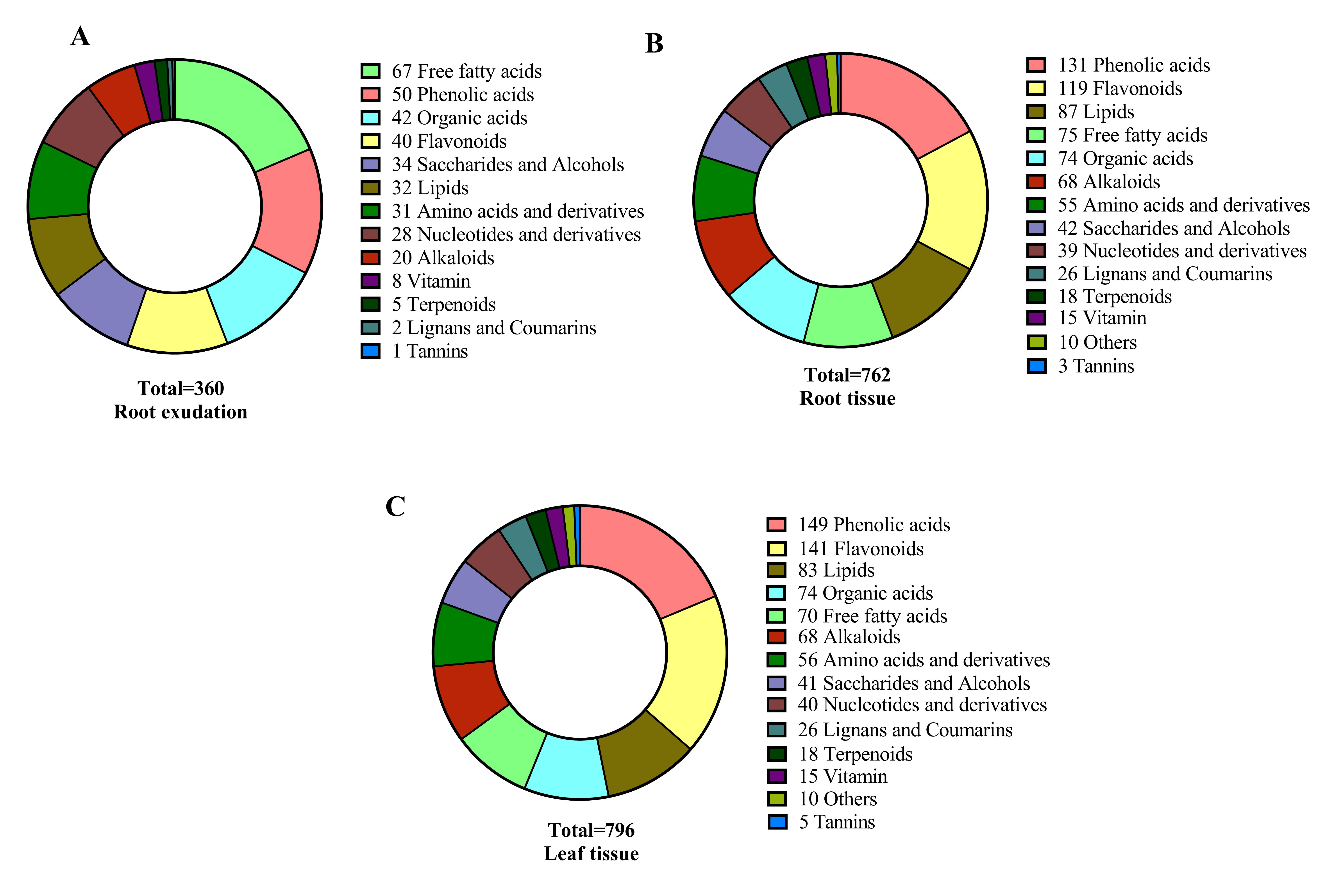

Supplement: Supplementary Figure 1 — Classification of the metabolites detected in root exudates (A), root tissues (B) and leaf tissues (C) of L. chinensis. [file Image_1.jpeg]

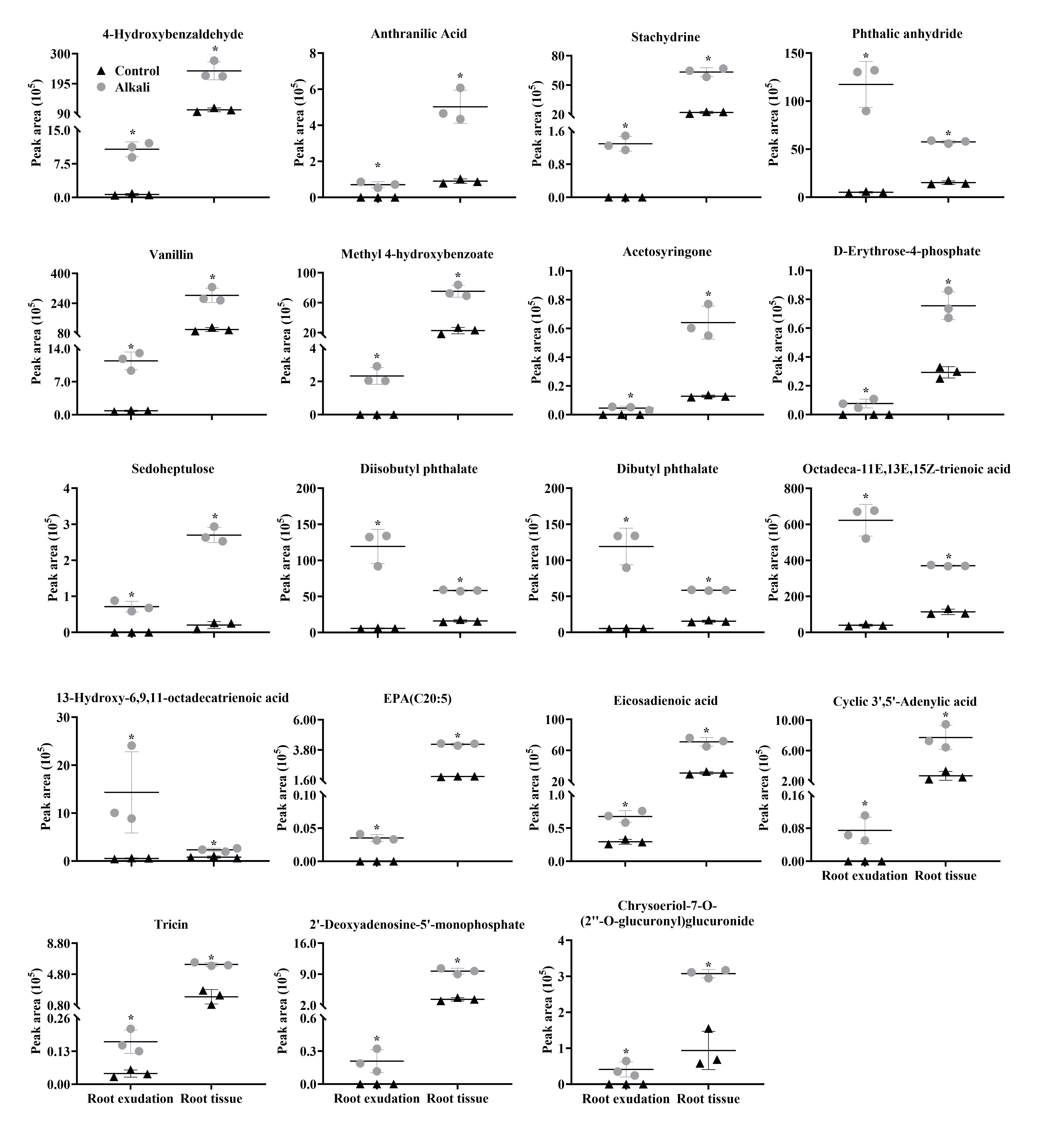

Supplement: Supplementary Figure 2 — Comparison of the effects of alkali stress on the accumulations of metabolites in root exudates and root tissues of L. chinensis. The levels of the metabolites were upregulated in both root exudates and root tissues. The asterisks (*) indicate significant differences between treatments. [file Image_2.jpeg]

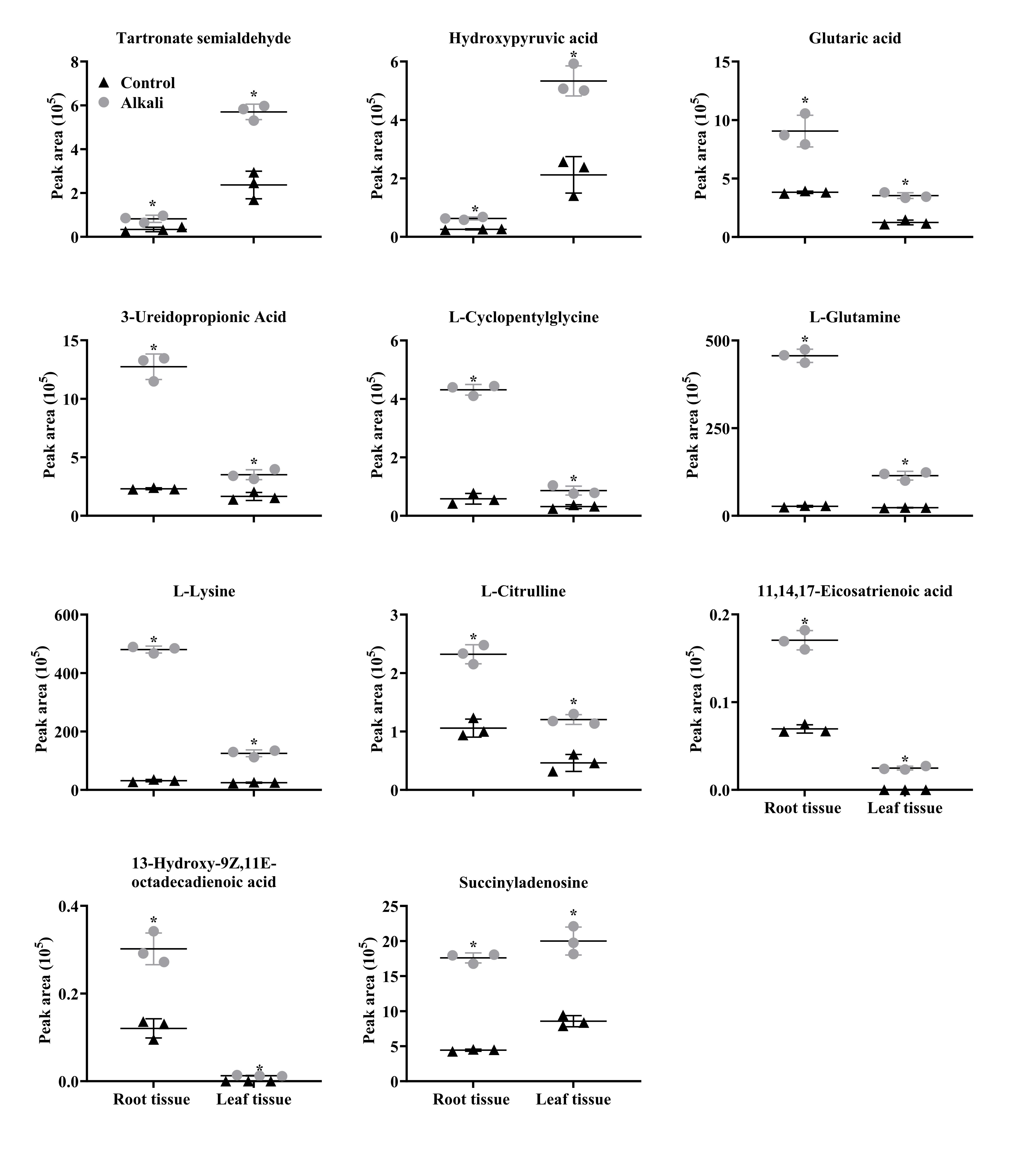

Supplement: Supplementary Figure 3 — Comparison of the effects of alkali stress on the accumulations of metabolites containing -COOH in roots and leaves of L. chinensis. The levels of the metabolites were upregulated in both roots and leaves. The asterisks (*) indicate significant differences between treatments. [file Image_3.jpeg]

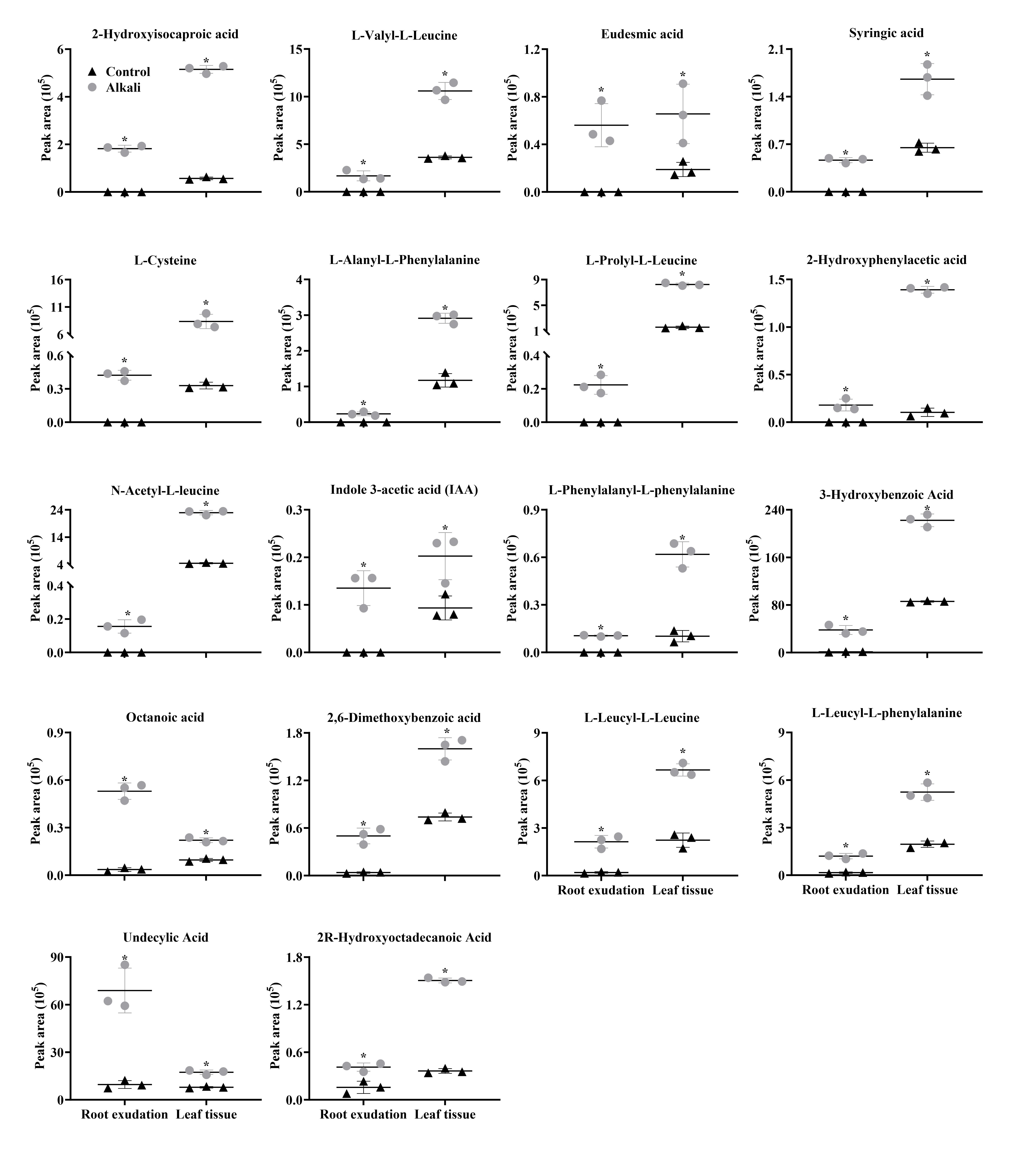

Supplement: Supplementary Figure 4 — Comparison of the effects of alkali stress on the accumulations of metabolites containing -COOH in root exudates and leaves of L. chinensis. The levels of the metabolites were upregulated in both root exudates and leaves. The asterisks (*) indicate significant differences between treatments. [file Image_4.jpeg]

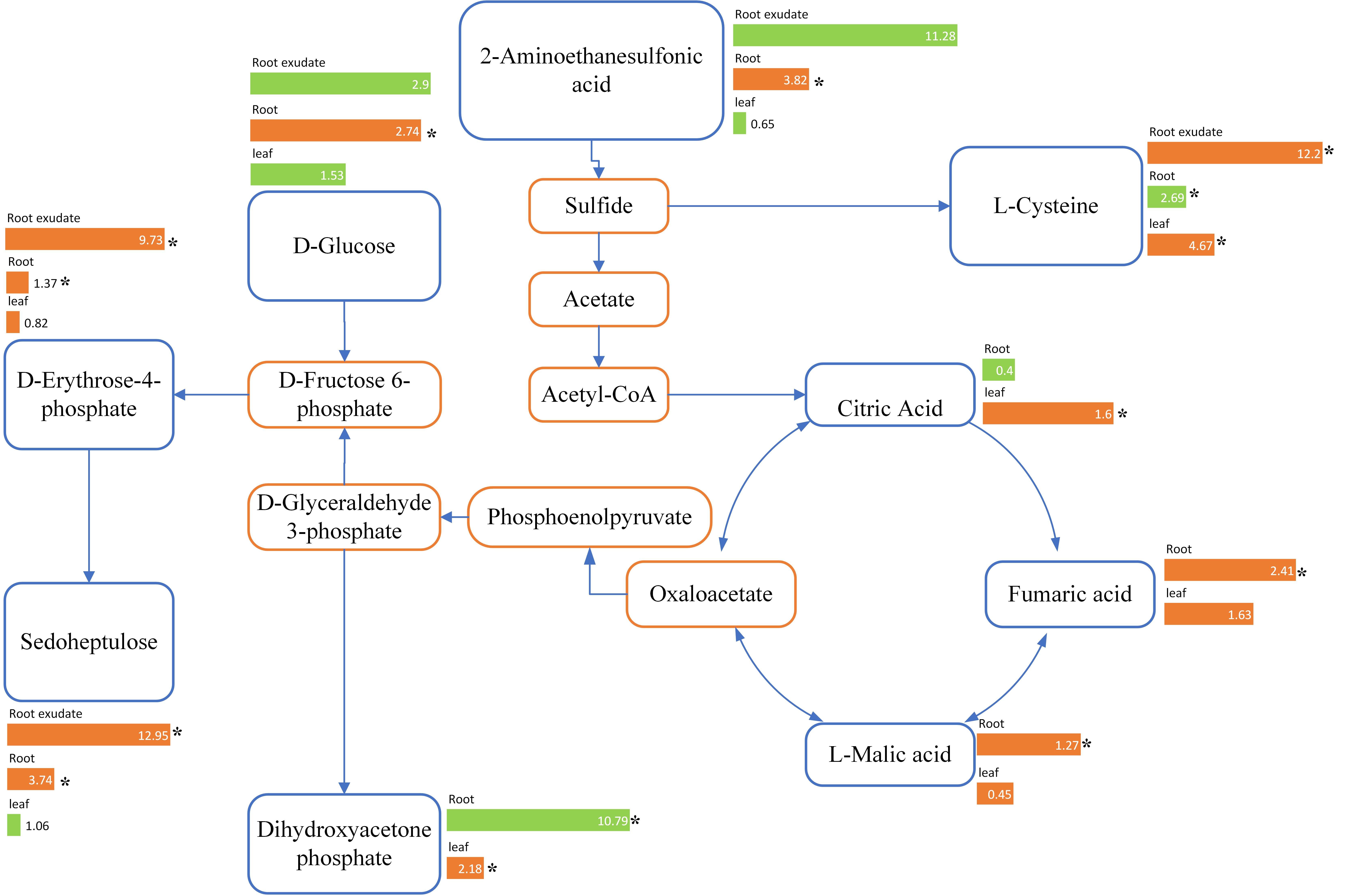

Supplement: Supplementary Figure 5 — Energy metabolism responses of root exudates, roots and leaves of L. chinensis under alkali-stress conditions. Red and green represent increased and decreased, respectively, accumulations of a metabolite. Yellow indicates that a metabolite was not detected. The asterisks (*) indicate significant differences between treatments. [file Image_5.jpeg]

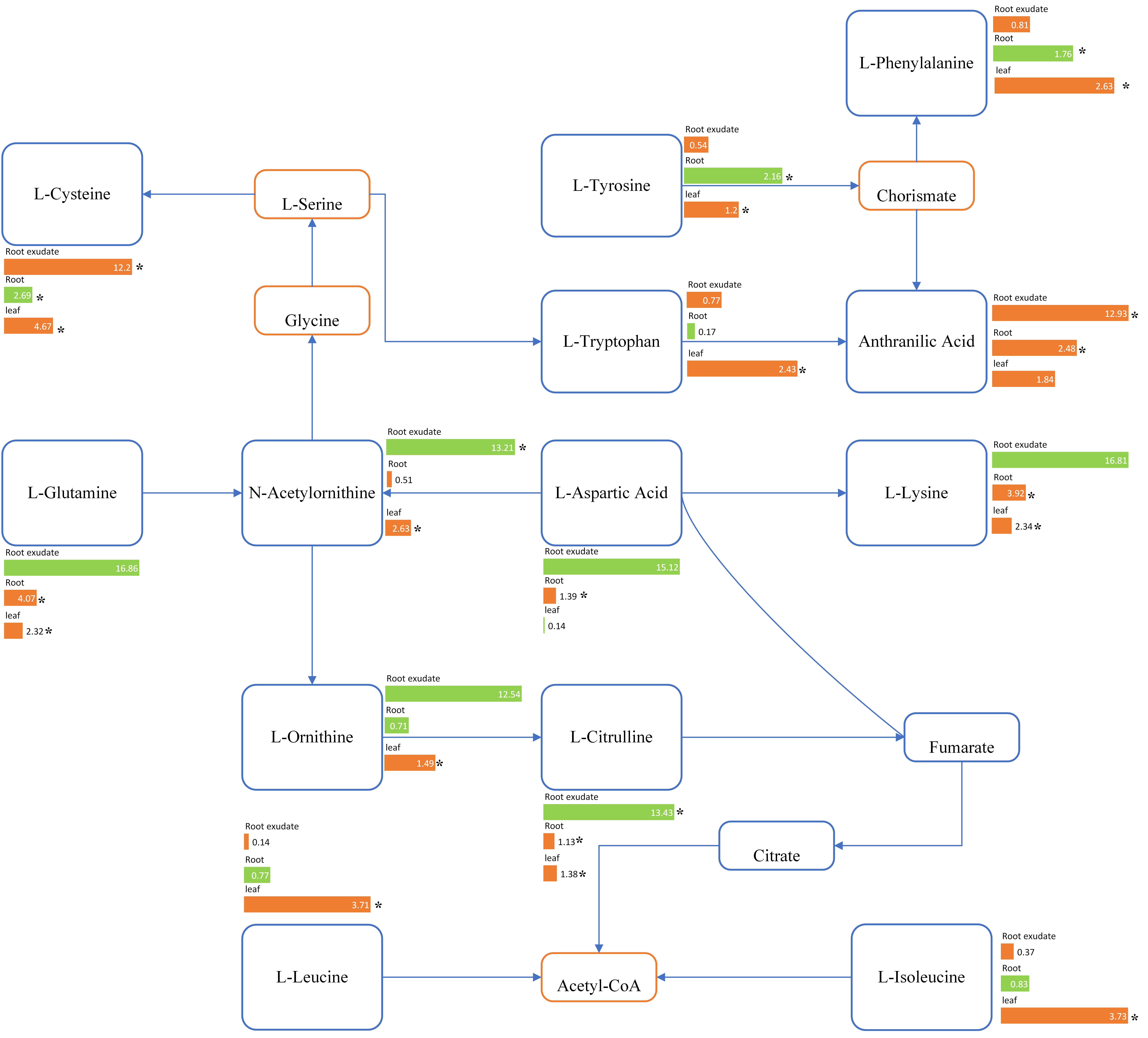

Supplement: Supplementary Figure 6 — Amino acid metabolism responses of root exudates, roots and leaves of L. chinensis under alkali-stress conditions. Red and green represent increased and decreased, respectively, accumulations of a metabolite. Yellow indicates that a metabolite was not detected. The asterisks (*) indicate significant differences between treatments. [file Image_6.jpeg]

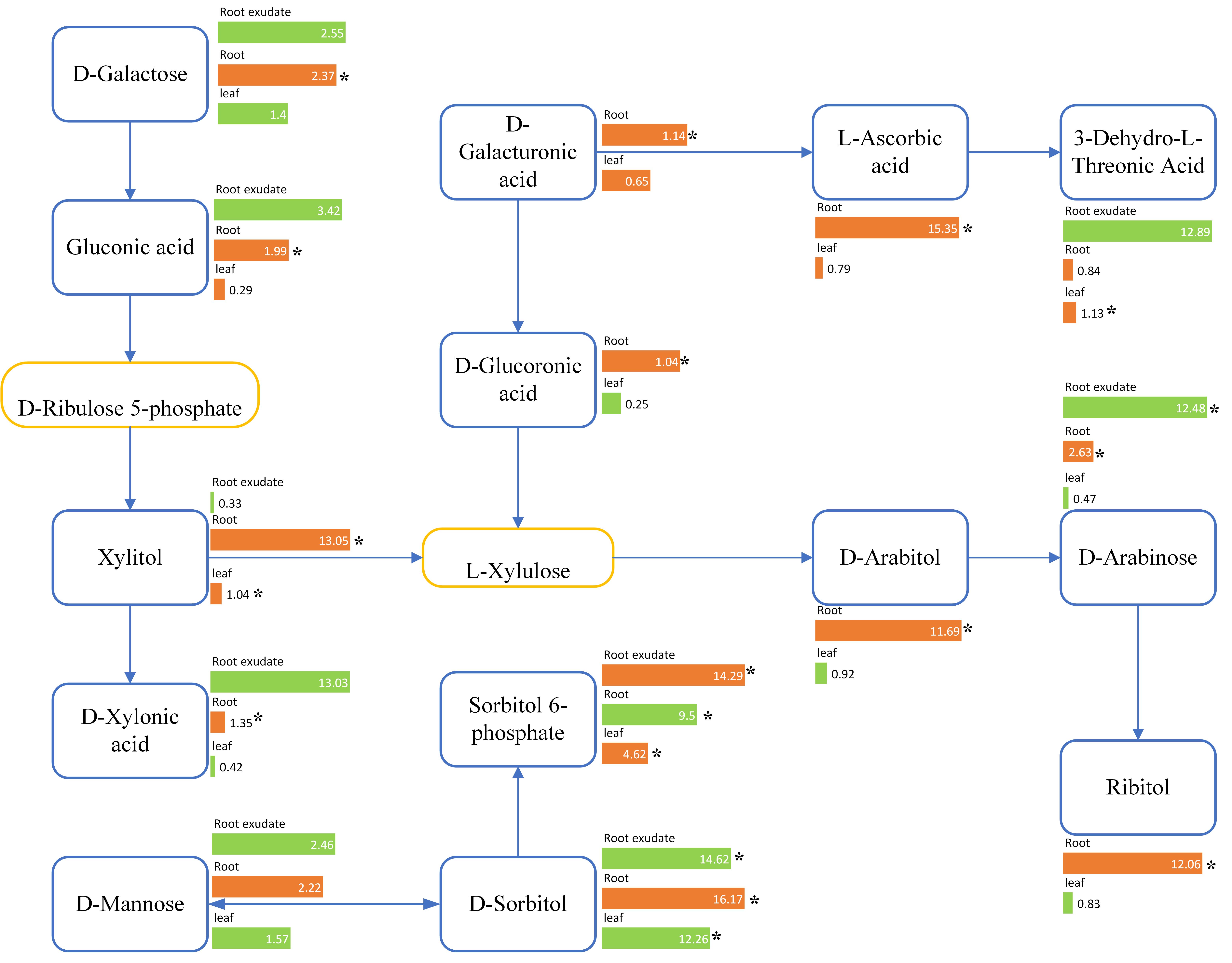

Supplement: Supplementary Figure 7 — Carbohydrate metabolism responses of root exudates, roots and leaves of L. chinensis under alkali-stress conditions. Red and green represent increased and decreased, respectively, accumulations of a metabolite. Yellow indicates that a metabolite was not detected. The asterisks (*) indicate significant differences between treatments. [file Image_7.jpeg]

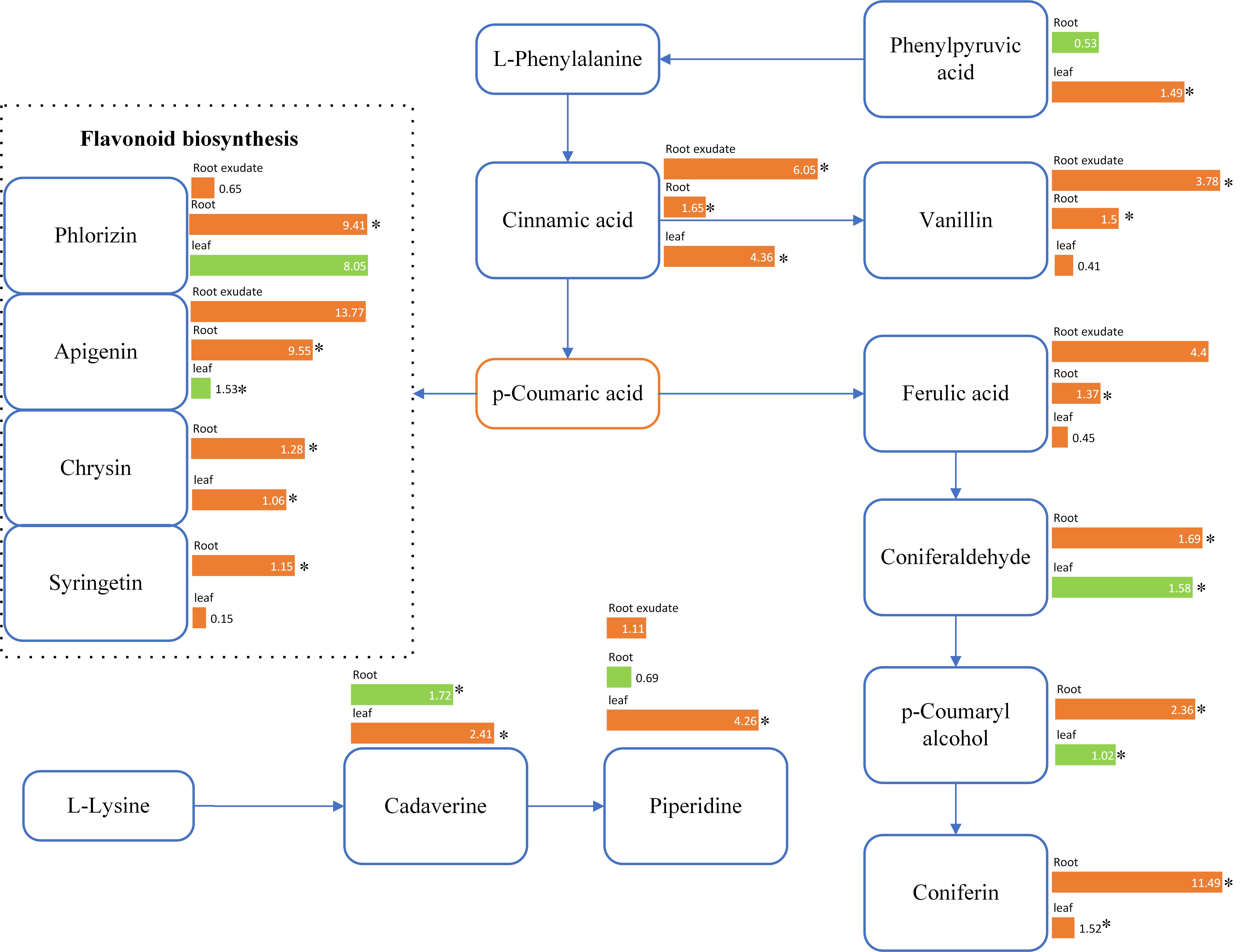

Supplement: Supplementary Figure 8 — Secondary metabolite responses of root exudates, roots and leaves of L. chinensis under alkali-stress conditions. Red and green represent increased and decreased, respectively, accumulations of a metabolite. Yellow indicates that a metabolite was not detected. The asterisks (*) indicate significant differences between treatments. [file Image_8.jpeg]
